# Prediction of Discharge Functional Independence Measure Scores From Routine Admission Variables Using a ChatGPT-Assisted Workflow in Patients With Cerebral Infarction or Intracerebral Hemorrhage: A Retrospective Observational Study

**DOI:** 10.7759/cureus.106640

**Published:** 2026-04-08

**Authors:** Isao Uno

**Affiliations:** 1 Rehabilitation Medicine, Sakurajyuji Fukuoka Hospital, Fukuoka, JPN

**Keywords:** convalescent rehabilitation ward, functional independence measure, large language models, rehabilitation, stroke

## Abstract

Background

Predicting discharge functional status is important in stroke rehabilitation, but developing local prediction models often requires statistical and programming expertise. The primary objective of this study was to develop and internally validate prediction models for discharge Functional Independence Measure (FIM) scores using routine admission variables. The secondary objective was to describe a locally executed ChatGPT-assisted workflow used only for template generation and code scaffolding.

Methods

This single-center retrospective observational study included 377 patients with cerebral infarction or intracerebral hemorrhage admitted to the convalescent rehabilitation ward, a Japanese post-acute inpatient rehabilitation unit for patients who require intensive rehabilitation after acute treatment, of Sakurajyuji Fukuoka Hospital (Fukuoka, Japan) between April 2022 and March 2025. Predictors were age, days from stroke onset to admission, and admission FIM scores. Separate linear regression models were developed for discharge motor FIM and discharge cognitive FIM, and a direct model was developed for discharge total FIM. Internal validation used five-fold cross-validation with out-of-fold predictions. Model performance was evaluated using the mean absolute error (MAE), root mean squared error (RMSE), coefficient of determination (R²), and calibration metrics.

Results

The discharge motor FIM model yielded an MAE of 11.87, an RMSE of 14.69, and an R² of 0.680. The discharge cognitive FIM model yielded an MAE of 3.76, an RMSE of 4.98, and an R² of 0.731. The direct discharge total FIM model yielded an MAE of 14.55, an RMSE of 18.21, and an R² of 0.714. Calibration slopes were close to 1.0 for all outcomes, and repeated cross-validation showed minimal variation in prediction error.

Conclusions

Discharge FIM scores were moderately predictable with good calibration using a small set of routinely available admission variables. A locally executed ChatGPT-assisted workflow was feasible for template generation and code scaffolding, but formal evaluation of clinical usefulness, transparent reporting, human verification, and external validation remain necessary.

## Introduction

Predicting functional status at discharge is important in stroke rehabilitation because it informs goal setting, discharge planning, and anticipated care needs. The Functional Independence Measure (FIM) is widely used in inpatient rehabilitation to quantify disability and recovery, and admission FIM has been associated with later care needs and discharge disposition after stroke [1-3].

Several studies have shown that admission functional status, age, and other clinical variables are associated with outcomes after stroke rehabilitation. Admission FIM has been reported as an independent predictor of good outcome, and admission total FIM has been identified as a strong predictor of discharge total FIM in inpatient stroke rehabilitation cohorts [4-6].

At the same time, recent work has shown that large language models can support research and data-analysis workflows through natural-language interaction and code-assisted analytics, while emerging reporting guidance emphasizes transparent disclosure, human oversight, and privacy protection when such tools are used in health research [7-10]. The primary aim of this study was to develop and internally validate regression models for discharge motor, cognitive, and total FIM scores using routinely available admission variables in patients with cerebral infarction or intracerebral hemorrhage admitted to a convalescent rehabilitation ward, a Japanese post-acute inpatient rehabilitation unit. The secondary aim was to describe the feasibility of a locally executed ChatGPT-assisted workflow used only for template generation and code scaffolding. We hypothesized that these routinely available admission variables would provide moderate predictive performance with good calibration.

## Materials and methods

Study design

This study was a single-center retrospective observational study of patients with cerebral infarction or intracerebral hemorrhage admitted to the convalescent rehabilitation ward, which in Japan is a post-acute inpatient rehabilitation unit for patients requiring intensive rehabilitation after acute treatment, of Sakurajyuji Fukuoka Hospital in Fukuoka, Japan.

Data source

Data were extracted from routinely collected information recorded in the medical charts. The study period was from April 2022 to March 2025.

Ethical considerations

This study was approved by the Ethics Committee of Sakurajyuji Fukuoka Hospital (approval number: 2025011402). Written informed consent was obtained from all participants. No patient-level data were shared with third parties.

Use of ChatGPT

ChatGPT (OpenAI, San Francisco, California, USA) was used on January 7, 2026, only to assist with drafting standardized input/output templates and code scaffolding for the planned analyses. No patient-level data were entered into ChatGPT. All generated code was reviewed by the author, revised as needed, and executed locally. Model fitting, validation, and performance evaluation were performed locally using structured clinical data. The final versioned analysis code used in this study is publicly available at GitHub release (https://github.com/isaouno/stroke-fim-prediction-2025/releases/tag/v1.0.1). The reporting of large language model use was aligned with emerging recommendations for transparent disclosure of tool specifications, human oversight, content verification, and privacy considerations [9-11].

Participants

The study population consisted of patients admitted to the convalescent rehabilitation ward with cerebral infarction or intracerebral hemorrhage. Eligible diagnoses were identified from the admission diagnosis recorded in the medical chart; in the source system, these diagnoses also corresponded to the administrative disease categories used for inpatient rehabilitation billing in Japan.

Patients were included in the analysis if complete data were available for age, days from stroke onset to admission, admission motor FIM, admission cognitive FIM, discharge motor FIM, and discharge cognitive FIM. The exclusion criteria were death, emergency transfer due to clinical deterioration, and missing data. The final analysis included 377 patients.

Variables

The predictors were age, days from stroke onset to admission, admission motor FIM, and admission cognitive FIM. Admission total FIM was calculated as the sum of admission motor FIM and admission cognitive FIM. The outcomes were discharge motor FIM, discharge cognitive FIM, and discharge total FIM. Discharge total FIM was calculated as the sum of discharge motor FIM and discharge cognitive FIM.

FIM assessment

FIM was assessed at admission and discharge by the therapist in charge of each patient. All evaluators had completed training on the FIM assessment procedure, and scoring was performed using a manual describing the scoring criteria.

Data quality control

Before analysis, the dataset was checked for missing or non-numeric values in all required variables. In addition, admission and discharge motor, cognitive, and total FIM scores were checked for values outside the allowable ranges. Internal consistency was also verified by confirming that admission and discharge total FIM scores were equal to the sum of the corresponding motor and cognitive FIM scores.

Handling of outliers

No observations were excluded as statistical outliers. Because all required variables fell within prespecified allowable ranges and no implausible values were identified during data quality checks, analyses were conducted on the full eligible sample without outlier-based deletion or winsorization.

Handling of missing data

A complete case analysis was performed. Patients with missing data in any variable required for the analysis were excluded. No missing values were present in the final analytic sample of 377 patients.

Prediction models

For discharge motor FIM and discharge cognitive FIM, separate multivariable linear regression models were developed using age, days from stroke onset to admission, admission motor FIM, and admission cognitive FIM as predictors. For discharge total FIM, a separate multivariable linear regression model was developed using age, days from stroke onset to admission, and admission total FIM as predictors. Because admission motor FIM, admission cognitive FIM, and admission total FIM are linearly dependent, only admission total FIM was used in the direct prediction model for discharge total FIM. In addition, a derived prediction of discharge total FIM was calculated as the sum of the predicted discharge motor FIM and predicted discharge cognitive FIM.

Statistical analysis

For internal validation, the analytic dataset was randomly partitioned into five approximately equal folds. In each iteration, the model was fitted on four folds and evaluated on the remaining fold, so that each patient contributed one out-of-fold prediction per repetition. Because the linear regression models and predictors were prespecified, no hyperparameter tuning or data-driven variable selection was performed within cross-validation. For the repeated cross-validation analysis, fold assignment was regenerated using different random seeds across 40 repetitions. Model performance was evaluated using mean absolute error (MAE), root mean squared error (RMSE), and coefficient of determination (R²).

Observed value = a + b × Predicted value,

where a represents the calibration intercept and b represents the calibration slope.

Ninety-five percent confidence intervals for performance and calibration indices were estimated using bootstrap resampling with 2,000 repetitions.

To evaluate prediction stability across the repeated cross-validation analyses, the mean and standard deviation of the MAE were calculated. In addition, the standard deviation of repeated predictions was calculated for each patient. Agreement among repeated predictions was assessed using ICC(2,1), and its 95% confidence interval was estimated using bootstrap resampling with 2,000 repetitions.

As a supplementary analysis, bootstrap optimism correction with 500 repetitions was performed.

Although integer rounding and range restrictions were considered as operational requirements for future implementation, these constraints were not applied in the present performance evaluation, and all predictions were assessed as continuous values.

Software

All statistical analyses were performed locally using Python 3.14.3 (Python Software Foundation, USA) and NumPy 2.4.0.

## Results

Study cohort

A total of 377 patients were included in the final analysis. The baseline characteristics of the study cohort are summarized in Table 1. No missing or non-numeric values were identified in the required variables. In addition, no out-of-range values were observed for admission or discharge motor, cognitive, or total FIM scores, and no discrepancies were found between total FIM scores and the sum of motor and cognitive FIM scores.

**Table 1:** Baseline characteristics of the study cohort (n = 377) Values are presented as mean ± standard deviation, median [interquartile range], and minimum–maximum. Total FIM scores were calculated as the sum of motor and cognitive FIM scores. FIM: Functional Independence Measure

Variable	n	Mean ± SD	Median (IQR)	Min–Max
Age, years	377	73.46 ± 14.71	76.00 (65.00–84.00)	20–104
Onset-to-admission days	377	30.72 ± 23.92	24.00 (17.00–35.00)	0–207
Admission mFIM	377	42.44 ± 18.84	47.00 (25.00–57.00)	13–89
Admission cFIM	377	22.96 ± 9.77	25.00 (15.00–32.00)	5–35
Admission total FIM	377	65.40 ± 26.53	70.00 (45.00–86.00)	18–124
Discharge mFIM	377	67.19 ± 25.99	79.00 (50.00–89.00)	13–91
Discharge cFIM	377	26.61 ± 9.61	31.00 (20.00–35.00)	5–35
Discharge total FIM	377	93.81 ± 34.11	109.00 (70.00–122.00)	18–126

Internal validation performance

The internal validation performance of the prediction models is presented in Table 2. In five-fold cross-validation using out-of-fold predictions, the model for discharge motor FIM achieved a mean absolute error (MAE) of 11.87, a root mean squared error (RMSE) of 14.69, and an R² of 0.680. The calibration intercept was 0.668, and the calibration slope was 0.990. The corresponding 95% confidence intervals were 11.02-12.79 for MAE, 13.68-15.73 for RMSE, 0.627-0.723 for R², -4.65 to 6.07 for the calibration intercept, and 0.919-1.062 for the calibration slope.

**Table 2:** Internal validation performance of the prediction models for discharge FIM outcomes Performance estimates are based on out-of-fold predictions from five-fold cross-validation. Ninety-five percent confidence intervals were estimated using bootstrap resampling (B = 2,000). Calibration was assessed by linear regression of observed values on predicted values (observed = a + b × predicted). The derived total FIM row was calculated as predicted motor FIM + predicted cognitive FIM. FIM: Functional Independence Measure

Model	Outcome	Predictors	MAE (95% CI)	RMSE (95% CI)	R² (95% CI)	Calibration intercept a (95% CI)	Calibration slope b (95% CI)	n
Model A	Discharge mFIM	Age + onset-to-admission days + admission mFIM + admission cFIM	11.87 (11.02–12.79)	14.69 (13.68–15.73)	0.680 (0.627–0.723)	0.668 (-4.648–6.068)	0.990 (0.919–1.062)	377
Model B	Discharge cFIM	Age + onset-to-admission days + admission mFIM + admission cFIM	3.76 (3.44–4.12)	4.98 (4.50–5.48)	0.731 (0.668–0.780)	0.433 (-1.375–2.437)	0.984 (0.924–1.041)	377
Model C	Discharge total FIM	Age + onset-to-admission days + admission total FIM	14.55 (13.46–15.65)	18.21 (16.80–19.56)	0.714 (0.664–0.758)	0.639 (-6.049–7.737)	0.993 (0.926–1.056)	377
Derived total	Predicted total FIM = predicted mFIM + predicted cFIM	Derived from Models A + B	14.47 (13.38–15.58)	18.17 (16.77–19.56)	0.716 (0.665–0.761)	1.065 (-5.755–8.132)	0.989 (0.924–1.054)	377

For discharge cognitive FIM, the model yielded an MAE of 3.76, an RMSE of 4.98, and an R² of 0.731, with a calibration intercept of 0.433 and a calibration slope of 0.984. The 95% confidence intervals were 3.44-4.12 for MAE, 4.50-5.48 for RMSE, 0.668-0.780 for R², -1.37 to 2.44 for the calibration intercept, and 0.924-1.041 for the calibration slope.

For discharge total FIM, the direct prediction model yielded an MAE of 14.55, an RMSE of 18.21, and an R² of 0.714, with a calibration intercept of 0.639 and a calibration slope of 0.993. The 95% confidence intervals were 13.46-15.65 for MAE, 16.80-19.56 for RMSE, 0.664-0.758 for R², -6.05 to 7.74 for the calibration intercept, and 0.926-1.056 for the calibration slope.

A derived total FIM score calculated as the sum of the predicted motor and predicted cognitive FIM scores also showed similar performance, with an MAE of 14.47, an RMSE of 18.17, and an R² of 0.716, with a calibration intercept of 1.065 and a calibration slope of 0.989. The 95% confidence intervals were 13.38-15.58 for MAE, 16.77-19.56 for RMSE, 0.665-0.761 for R², -5.76 to 8.13 for the calibration intercept, and 0.924-1.054 for the calibration slope.

Stability and sensitivity analyses

The stability and sensitivity analyses are summarized in Table 3. Across 40 repeated five-fold cross-validations, the mean ± standard deviation of the MAE was 11.80 ± 0.07 for discharge motor FIM, 3.71 ± 0.02 for discharge cognitive FIM, and 14.47 ± 0.07 for discharge total FIM.

**Table 3:** Stability and sensitivity analyses of the prediction models Repeated cross-validation results are based on 40 repetitions of five-fold cross-validation using different random seeds. Per-subject prediction SD refers to the standard deviation of repeated out-of-fold predictions for each participant. ICC(2,1) 95% confidence intervals were estimated by bootstrap resampling (B = 2,000). Optimism-corrected performance was obtained using bootstrap optimism correction (B = 500). FIM: Functional Independence Measure

Model	Outcome	Repeated CV MAE, mean ± SD	Per-subject prediction SD, median [IQR]	ICC(2,1) (95% CI)	Optimism-corrected MAE	Optimism-corrected RMSE	Optimism-corrected R²
Model A	Discharge mFIM	11.80 ± 0.07	0.753 [0.589–0.945]	0.998397 (0.998181–0.998581)	11.80	14.63	0.684
Model B	Discharge cFIM	3.71 ± 0.02	0.253 [0.189–0.325]	0.998713 (0.998512–0.998882)	3.69	4.90	0.741
Model C	Discharge total FIM	14.47 ± 0.07	0.817 [0.628–1.027]	0.998911 (0.998753–0.999038)	14.45	18.13	0.718

The median per-subject standard deviation of repeated predictions was 0.753 (interquartile range (IQR), 0.589-0.945) for discharge motor FIM, 0.253 (IQR, 0.189-0.325) for discharge cognitive FIM, and 0.817 (IQR, 0.628-1.027) for discharge total FIM.

The intraclass correlation coefficient, ICC(2,1), was 0.998397 (95% CI, 0.998181-0.998581) for discharge motor FIM, 0.998713 (95% CI, 0.998512-0.998882) for discharge cognitive FIM, and 0.998911 (95% CI, 0.998753-0.999038) for discharge total FIM.

After bootstrap optimism correction, the corrected performance estimates were as follows: for discharge motor FIM, MAE 11.80, RMSE 14.63, and R² 0.684; for discharge cognitive FIM, MAE 3.69, RMSE 4.90, and R² 0.741; and for discharge total FIM, MAE 14.45, RMSE 18.13, and R² 0.718.

Final model equations

The full regression equations fitted on the complete analytic dataset were as follows:

Predicted discharge motor FIM = 39.241 - 0.238 × age - 0.096 × onset-to-admission days + 0.826 × admission motor FIM + 0.581 × admission cognitive FIM.

Predicted discharge cognitive FIM = 13.663 - 0.073 × age - 0.020 × onset-to-admission days + 0.031 × admission motor FIM + 0.767 × admission cognitive FIM.

Predicted discharge total FIM (direct model) = 53.550 - 0.306 × age - 0.115 × onset-to-admission days + 1.013 × admission total FIM.

## Discussion

In this single-center retrospective study, regression models based on routinely available admission variables showed moderate predictive performance and good calibration for discharge motor, cognitive, and total FIM outcomes. ChatGPT was used only to assist with template generation and code scaffolding, and no patient-level data were entered into the model. Calibration slopes were close to 1.0 for all three outcomes, and repeated cross-validation showed minimal variation in MAE, with ICC(2,1) values exceeding 0.998. Taken together, these findings suggest that discharge functional status can be moderately predicted using only a small set of routinely available admission variables [2-5].

A clinically important finding is that moderate predictive performance with good calibration was obtained from four simple admission variables: age, days from stroke onset to admission, admission motor FIM, and admission cognitive FIM. These variables are available early in routine care and do not require additional testing, which may support early discharge planning and communication with patients and families, although clinical usefulness was not formally quantified in this study. Prior studies have shown that admission FIM is associated with later care needs and discharge placement after stroke [2,3], and that admission functional status is a strong predictor of rehabilitation outcome [4,5]. Our findings support the feasibility of predicting discharge motor, cognitive, and total FIM in a convalescent rehabilitation setting using a parsimonious set of predictors.

In this study, ChatGPT was not used to infer outcomes directly from patient-level data. Instead, it was used to generate the analytic workflow and executable code, while model fitting, validation, and performance assessment were performed locally using structured clinical data. This distinction is important because the value of a large language model in this context may lie in supporting technical implementation while preserving the need for statistical verification and human oversight [7].

These findings should be interpreted in light of prior stroke rehabilitation prediction research. A systematic review by Meyer et al. found that admission functional level and age were among the predictors most consistently associated with functional outcome after post-stroke inpatient rehabilitation [12]. Sonoda et al. likewise reported that age, days from stroke onset to admission, and admission motor and cognitive FIM contributed to the prediction of discharge motor FIM in a rehabilitation cohort [13]. In a machine-learning study of inpatient stroke rehabilitation, Harari et al. reported that admission clinical test scores were the most important predictors of discharge outcomes and that time from stroke onset to rehabilitation admission and age also contributed to prediction performance [14]. More recently, Campagnini et al. developed internally cross-validated and externally validated machine-learning models for post-stroke inpatient rehabilitation and found that baseline motor and cognitive measures and age were among the main contributors to outcome prediction [15]. Taken together, these studies and the present results support the importance of readily available admission functional data and age while also highlighting that transparent reporting and external validation remain essential for clinical adoption [12,15-17].

Several limitations should be acknowledged. First, this was a single-center retrospective study, and external validation was not performed. Therefore, the generalizability of the models to other institutions and other patient populations remains unknown [12,17]. Second, because the analysis was restricted to patients with complete data who completed the rehabilitation course to discharge, selection bias cannot be excluded. Patients who died, required emergency transfer, or had missing data may have differed systematically from the analyzed cohort in severity or recovery trajectory. Third, although all evaluators had completed FIM training and used a scoring manual, formal inter-rater reliability was not assessed within this dataset; therefore, residual measurement variability may have influenced model performance. Fourth, the number of candidate patients and the detailed exclusion breakdown were not available for reporting. Fifth, the models were intentionally parsimonious and did not include other potentially relevant predictors, which may improve performance in future studies but would also increase data collection burden [12,16]. Finally, we did not compare the prespecified regression models with simpler benchmark models or alternative machine-learning approaches, and we did not directly compare the ChatGPT-assisted workflow with a conventional non-LLM analytic workflow.

Despite these limitations, the present study has practical implications. The combination of a parsimonious admission dataset, local execution without sharing patient-level data with an external model, favorable internal validation metrics, and publicly released versioned code supports the feasibility of an AI-assisted workflow for local prediction-model development in rehabilitation settings. However, the present study did not formally quantify clinical utility and does not establish superior efficiency or performance of either the selected modelling strategy or the AI-assisted workflow. Future studies should perform external validation in independent cohorts, compare the prespecified regression models with simpler benchmark models and alternative approaches, directly compare the direct total-FIM model and the derived total-FIM approach based on predicted motor and cognitive FIM, and evaluate whether adding clinically meaningful predictors improves performance without substantially reducing feasibility. Transparent reporting of AI-assisted prediction model studies, including disclosure of tool specifications, human oversight, verification procedures, privacy considerations, and code availability, will also be important for appraisal and implementation [9-11].

## Conclusions

In patients with cerebral infarction or intracerebral hemorrhage admitted to a convalescent rehabilitation ward, discharge motor, cognitive, and total FIM scores were moderately predictable with good calibration using only routinely available admission variables. In this study, ChatGPT was used only for template generation and code scaffolding under human review and local execution. External validation, comparison with benchmark models, and formal evaluation of clinical usefulness are needed before implementation.
